# LivRelief varicose veins cream in the treatment of chronic venous insufficiency of the lower limbs: A 6-week single arm pilot study

**DOI:** 10.1371/journal.pone.0208954

**Published:** 2018-12-31

**Authors:** Heather C. Dwyer, David C. Baranowski, Perry V. Mayer, Simona Gabriele

**Affiliations:** 1 Research & Development Department, Delivra Inc; Hamilton, Ontario, Canada; 2 Research & Development Department, Delivra Inc, Charlottetown, Prince Edward Island, Canada; 3 The Mayer Institute, Hamilton, Ontario, Canada; 4 Department of Medical Science, McMaster University, Hamilton, Ontario, Canada; The Second Affilated Hospital, Zhejiang University School of Medicine, CHINA

## Abstract

**Background:**

Chronic Venous Disease is characterized by morphological abnormalities of the venous system. Affected limbs are classified in increasing clinical severity with the Clinical Etiological Anatomical and Pathological system from C0 to C6. Limbs assessed at C3 through C6 meet the criteria of Chronic Venous Insufficiency. Chronic Venous Insufficiency of the Lower Limbs is a very common pathology affecting approximately ~40% of the world’s population. This study observes the use of the LivRelief Varicose Vein Cream, a Natural Health Product that is licensed for sale by Health Canada, for use in the treatment of varicose veins.

**Methods:**

An open label, single arm interventional, pilot study was conducted to determine the feasibility of recruitment and data collection in this population. To accomplish this, the cream was provided to all enrolled subjects. Subsequently, objective and subjective measures were performed at baseline and after 6 weeks of at-home use. Recruitment and data collection targets of at least 70% were established and the data collected at both timepoints were compared and analyzed using a paired t-test. Results were also reported as proportions where appropriate.

**Results:**

A total of 32 subjects were enrolled. The pre-defined feasibility objectives for recruitment and data collection were met with the enrolment of 97% of all screened patients and the collection of 94% of all scheduled data. The most significant therapeutic improvement was seen in the results of the Venous Clinical Severity Score where 66% of the treated legs experienced a decrease in severity after 6 weeks of treatment. P values were *<*0.0001 and 0.0003 for the left and right leg, respectively.

**Conclusion:**

It is feasible to recruit and collect data with the chosen outcome assessments within this population. Preliminary results suggest that the product could improve some of the clinical symptoms associated with the presence varicose veins. These results warrant further exploration in a longer, randomized and placebo-controlled study.

**Trial registration:**

Clinicaltrial.gov: NCT03653793.

## Introduction

Chronic venous disease (CVD) can be of either primary and secondary etiology [1]. Primary CVD is characterized by morphological and functional abnormalities of the venous system, wherein these changes are idiopathic (i.e. of unknown origin). In contrast, secondary CVD is often the result of venous thrombosis that leads to localized obstruction and/or reflux that generate similar morphological deformations [1–5]. A CVD diagnosis is confirmed and classified with the Clinical, Etiologic, Anatomical, and Pathophysiological (CEAP) assessment [1]. The CEAP was developed during the 6^th^ annual meeting of the American Venous Forum (AVF) in 1994. It was then universally accepted and endorsed by the councils of the Society for Vascular Surgery and the North American Chapter of the International Society for Cardiovascular Surgery. Its basic elements were then incorporated in venous reporting standards and continues to be widely referenced in publications on CVD [6]. Intra-observer and interobserver variability in classification led to the revision of the CEAP in 2004 and later the refinement of the classification system [6].

Clinical signs of venous disease that manifest in the legs are categorized according to the symptoms experienced, which include: tingling, aching, burning, pain, muscle cramps, sensations of swelling, throbbing or heaviness, itching skin, restless legs, leg tiredness and/or fatigue. The categories/classes within the CEAP increase in disease severity from C0: no visible or palpable signs of venous disease to C6: active venous ulcers [1, 7]. Legs that meet the criteria of each class may be symptomatic or asymptomatic [1, 7]. The term chronic venous insufficiency (CVI) implies functional abnormality of the venous system and is usually reserved for the more severe cases, classes C3 to C6 [1, 6]. Varicose veins, a characteristic of C2 that may be seen in limbs of higher classification, are tortuous or twisted veins that may be visibly enlarged or physically protrude due to the engorgement of blood within the peripheral lower limb vasculature [6]. This pathological condition is estimated to be one of the most common in adults within industrialized countries, affecting ~40% of the population [8]. Mechanically, this deformation and poor blood flow is related to the reduction in tensile strength and function of vein leaflets that typically ensure a unidirectional movement of blood [9]. The loss of leaflet function causes blood reflux and leads to localized pooling in the varicose veins.

Compression therapy is the cornerstone/mainstay of conservative treatment for CVD and considered first-line therapy for patients with symptomatic varicose veins [10–14]. The objective of compression therapy is to provide graded compression to the leg and oppose the hydrostatic forces of venous hypertension. Compression garments have been shown to reduce the residual volume and reflux in vein segments and may result in significant improvement in pain, swelling, skin pigmentation, patient activity and well being if compliance is maintained between 70–80% [10]. Other important and generally prescribed forms of conservative therapy include: leg elevation (where possible)[15, 16],pharmacologic therapy/vasoactive drugs (VAD) and exercise therapy [10, 11]. VADs include coumarins, flavonoids, saponosides and other plant extracts which improve venous tone and capillary permeability [10]. Exercise therapy improves calf muscle function. Its contraction forces blood out the venous plexi and up the deep venous system of the legs which may be beneficial in advanced disease treatment when used as a supplement to surgical intervention [10]. If conservative/non-invasive measures yield unsatisfying results, invasive interventions are pursued based on the anatomical and pathophysiological features of the diagnosis[10]. These include: endovenous deep system therapy, surgery for truncal vein or venous tributaries, perforator vein surgery and valve reconstruction [10]. Less invasive interventions include: foam sclerotherapy, endovenous thermal and radio frequency ablation [11].

While the CEAP is the clinical standard for disease confirmation and classification, it is not ideal for assessing therapeutic efficacy due to the static nature of the measurements in each class, particularly C4 and C5 [17, 18]. To address this need, the Venous Clinical Severity Score (VCSS) was created as a complement to the CEAP with the ability to generate a dynamic score and provide additional sensitivity within the CEAP C4 –C6 range. Validity and reliability of the VCSS has been established. Scores are consistent with a CEAP diagnosis, with favorable intra-observer variability and reliability coefficients [19]. A 30-point scoring instrument, the VCSS was developed by the American Venous Forum with nine clinical characteristics that are most indicative of therapeutic changes. It also has a tenth category for the use of compression garments and all categories are scored from 0 to 3[19]. The difference in sensitivity between the CEAP and VCSS in response to therapy is illustrated in a comparison of results from both tests conducted in this study [17].

The physician’s evaluation of clinical symptoms using tools like the VCSS, is one of the two ways that the efficacy of CVD therapy can be evaluated. The other is the use of patient perceived Quality of Life (QoL) measurements [16]. QoL instruments may be either generic or disease specific. Generic tools are appropriate for a wide range of disease states because they assess a global state of well being and provide a subjective measure of treatment efficacy. They also facilitate a comparison between therapeutic areas and different studies. The Q-LES-Q-SF and the Nottingham Health Profile are examples of validated generic QoL instruments [17, 20–27]. By comparison, disease specific instruments are more sensitive to therapeutic change because the surveys focus on the disease process and outcomes indicative of disease severity. This method of assessing the condition of interest has become more popular in venous disease reporting. Examples of such instruments are: The Chronic Venous Insufficiency Questionnaire (CIVIQ -20), Venous Insufficiency Epidemiological and Economic Study, Aberdeen Varicose Vein Questionnaire (AVVQ) and the Charing Cross Venous Ulceration Questionnaire[17].

Edema, a perceptible fluid increase in the skin and tissues that may extend from the ankle to the leg [7], is a common sign of CVD and a characteristic of C3 [7, 28]. As a result, it is often used as an indirect measure of severity in clinical studies and may be measured as changes in leg volume, leg circumference, opto-electronic volumetry and with various imaging options [28]. Using the assessments previously described, it is anticipated that therapeutic efficacy in the treatment of CVD can be demonstrated with a clinically meaningful decrease in VCSS and leg circumference, as well as an equally significant increase in QoL score.

Within Canada the regulation of non-surgical therapy for varicose veins can be segmented into two regulatory divisions, the Therapeutic Products Directorate (TPD) that oversees drug and medical device usage and the Natural and Non-prescription Health Products Directorate (NNHPD) that oversees products deemed to originate from natural sources rather than human-derived innovations [29]. The NNHPD acknowledges that topical witch-hazel (*Hamamelis virginiana*) [30], oral horse-chestnut (*Aesculus hippocastanum*) seed extract [31], and oral rutin [32] have vasoactive properties, that may help treat varicose veins, chronic venous insufficiency and protect blood vessels, respectively.

A Natural Product License, with the issuance of a Natural Product Number (NPN), is required for the sale of natural health products in Canada. Depending on the type of application, the submission of the relevant Natural and Non-Prescription Health Product Directorate (NNHPD) monograph may be required. Other references may be required depending on the type of application, some of which require the submission of safety and evidence reports [33]. Delivra Inc. currently holds a Natural Product license for the manufacturing and sale of topical LivRelief^TM^-Varicose Veins Cream. It has the Natural Product Number (NPN) 80029345) and contains 10% (w/w) witch-hazel as the active ingredient in a formulation that also includes 2% horse chestnut and 2% rutin. This study was conducted as a feasibility pilot for a future randomized control clinical trial and to gather preliminary data on patient reported efficacy. To accomplish this, Delivra Inc. completed an interventional pilot study within the scope and limitations of the existing product license.

## Materials and methods

### Study design

Patients of The Mayer Institute, that met the study’s eligibility criteria (Table 1) were recruited during routine clinic visits on the following dates: May 23 and 25, 2017 and June 23 and 30, 2017. All subjects were followed for a minimum of 6-weeks. The last study visit occurred on August 28, 2017. Prior to screening, written informed consent was obtained from interested patients by a study coordinator. A copy of the signed consent form was then provided to each subject. This study was retrospectively registered on 31 August 2018 at clinicaltrials.gov (NCT03653793) due to the authors’ ignorance of this requirement for a non-randomized clinical trial. However, any future studies involving human subjects will be registered prospectively. A preliminary dataset (N = 30) was proposed to evaluate product use and the quality of collected data. However, the study was not powered to detect a statistically significant treatment effect.

**Table 1:** Eligibility criteria.

Inclusion Criteria
• Adults: ≥ 19 years of age.
• Presence of lower limb varicose veins.
Exclusion Criteria
• Allergy to Witch-Hazel or other ingredients of the cream.
• Intent to undergo surgical treatment for varicose veins within the six weeks of study participation.
• Pregnant, breastfeeding or planning to become pregnant within the six weeks of study participation.
• Any dementia or major cognitive dysfunction that would preclude the individual’s ability to provide informed consent or complete the case report form.
• Any unstable medical condition (including but not limited to cardiovascular, cardiac/hypertension disease, moderate to severe kidney disease and moderate to severe liver disease).
• Any medical condition that would preclude the participant’s or a caregiver’s ability to administer the product daily for the duration of the study.
• An active ulcer at the site of product application (as evaluated during the CEAP assessment performed at screening.

Enrolled subjects were provided a 6-week supply of varicose vein cream for out-patient use and instructed to use the product as directed on the packaging, “1 pump (0.8ml) applied to the affected area twice daily”. Five assessments were performed at baseline (visit 1) and at the end of 6-weeks of twice daily use (visit 2). Both visits were conducted at The Mayer Institute and each subject was assessed by the same research nurse at both visits. The CEAP [34, 35], Venous Clinical Severity Score (VCSS) [35, 36], photography of the treated leg and circumferential measurements of the treated limbs were all performed by the study nurse. The circumferential measurements were performed at baseline and at the end of treatment to determine if there was a change in peripheral edema. This assessment was executed in accordance with the study specific SOP to ensure consistency between subjects and visits. The SOP included anatomical diagrams and a clear definition of the points to be measured. A tape measure was used to measure the circumference of the mid foot with a diameter of the styloid process by the base of the 5^th^ metatarsal to the navicular region on the opposite side of the foot. The media malleolus was described as a bony protrusion. The apex of the media malleolus was located and a tape measure used to measure 5cm upwards towards the knees. The circumference of the ankle was then measured at the 5cm mark. The apex of the tibial tuberosity was located, a tape measure was used to measure 5cm downwards towards the ankles. At the 5cm mark the circumference of the leg was measured. The Q-LES-Q-SF [26] was self administered by subjects. Participants were also asked to state if they were using compression garments at each visit. The principal investigator (PI) reviewed the collected screening data prior to confirming eligibility and the entire case report form (CRF) was reviewed by the study coordinator for completion at the end of each visit, prior to subject discharge.

A modified version of the VCSS questionnaire was used in this study. It collected results for 9 of the 10 original questions. Post-hoc quantification of compression garment usage was also performed. All participants were asked if they used compression garments at each visit and a “Yes” or “No” response was recorded. Usage was assumed to be bilateral and compliance with usage was not confirmed. All “Yes” and “No” responses were entered as “1” (intermittent use of stocking) and “0” (no use of garments), respectively. No patient was assigned “2” (wears stockings most days) or “3” (full stocking compliance). The response to this question on compression stocking usage replaced question 10 of the official VCSS. Protocol deviations have been listed in Table 2.

**Table 2:** Summary of protocol deviations.

Subject ID	Deviation description
01–001	A pregnancy test is included in the list of screening procedures. However, the test was not performed for this patient who was post-menopausal at Visit 1.
01–003	Q-LES-Q-SF–The subject did not answer one question in the assessment at Visit 1. The test was invalid and the subject omitted from analysis.
01–008	Q-LES-Q-SF–The subject did not answer one question in the assessment at Visit 2. The test was invalid and the subject omitted from analysis.
01–010	Early termination of treatment. Subject used the cream for 5 weeks and not 6 weeks because the pump dispenser of the bottle was defected.
01–019	The subject was unable to attend Visit 2 as scheduled. This visit was rescheduled and performed 6 days late (3 days out of window).
01–026	The subject was unable to attend Visit 2 as scheduled. This visit was rescheduled and performed 17 days late (2 weeks out of window).
01–032	Early termination of treatment after 2 days of use due to the occurrence of an adverse event.

### Outcomes

The primary outcome of the study was feasibility with the following objectives: 1). Recruit ≥ 70% of all eligible patients and, 2). Collect all planned data for ≥ 70% of all subjects. An intention to treat (ITT) concept was used in the post hoc exploratory analysis. Outcomes included significant differences between the results of assessments performed at baseline and those obtained at the end of treatment. Statistical analysis of the VCSS, Q-LES-Q-SF and circumferential measurements of the treated legs was performed using a paired t-test with the results of the left and right leg reported independently. Proportions were reported for the CEAP and skin discoloration assessments.

### Ethics approval and consent to participate

The study was conducted in accordance with Good Clinical Practice (GCP) and reviewed and approved by Institutional Review Board (IRB) services, protocol number (Pro00020990), prior to study commencement. Refer to submitted study protocols with ethics approvals S1 Protocol.

## Results

### Feasibility

**Fig 1:**
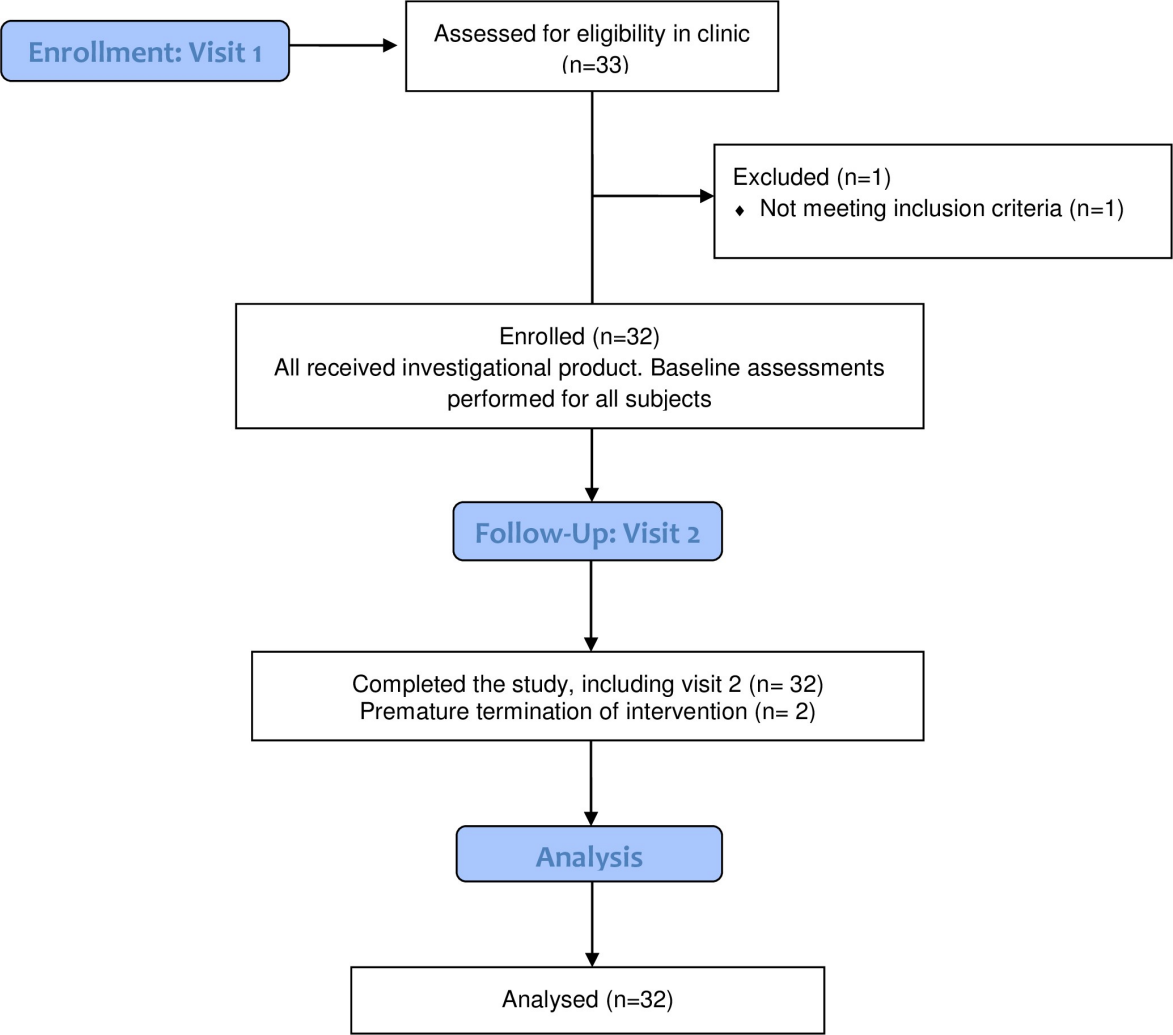
Study plan and subject accountability.

The study met both feasibility objectives of the primary outcome. Specifically, 32 of the 33 patients screened (97%) were successfully enrolled and complete dataset collection was achieved for 30 of the 32 subjects, i.e.: 94%. The sample consisted of 32 “Non-Hispanic/Non-Latino” participants, of which 6 were females and 26 were males. The average age of the group was 69.5 years, ranging from 52–83 years. It should be noted that 30 of the 32 subjects were diabetic, reflecting the increased incidence of vascular abnormalities associated with this pathophysiology. 31 subjects completed treatment as scheduled and one subject prematurely discontinued treatment due to a non-serious adverse event (see Fig 1). A CEAP exam was performed as part of the screening process to confirm the presence of varicose veins at baseline. The clinical profile of the sample of 64 legs at baseline was: C4a = 89%, C4b = 6%, C3 = 2% and C1 = 3%.

### Efficacy

Data collected at baseline for each subject/ affected limb was compared to data collected at the end of 6 weeks of scheduled treatment.

#### VCSS

The VCSS assessment was administered by the same research nurse at both Visit 1 and visit 2 (Table 3.)

**Table 3:** Venous Clinical Severity Score (VCSS) and statistical evaluation: Before and after 6 weeks of treatment with the varicose vein cream.

	Visit -1		Visit -2		Inter-visit p-values†	
	Right Leg	Left Leg	Right Leg	Left Leg	Right-Right	Left-Left
					(V1 to V2)	(V1 to V2)
Mean Score	9.9	10.1	8.1	7.9	0.0003	<0.0001
STDEV	2.8	3.2	1.9	1.9		
SEM	0.5	0.6	0.3	0.3		

Averaged values along with standard deviations and standard errors of the mean (SEM) are described along with statistical analysis using a paired t-test. P-values are described for inter-visit comparison for effective change (†).

**Fig 2:**
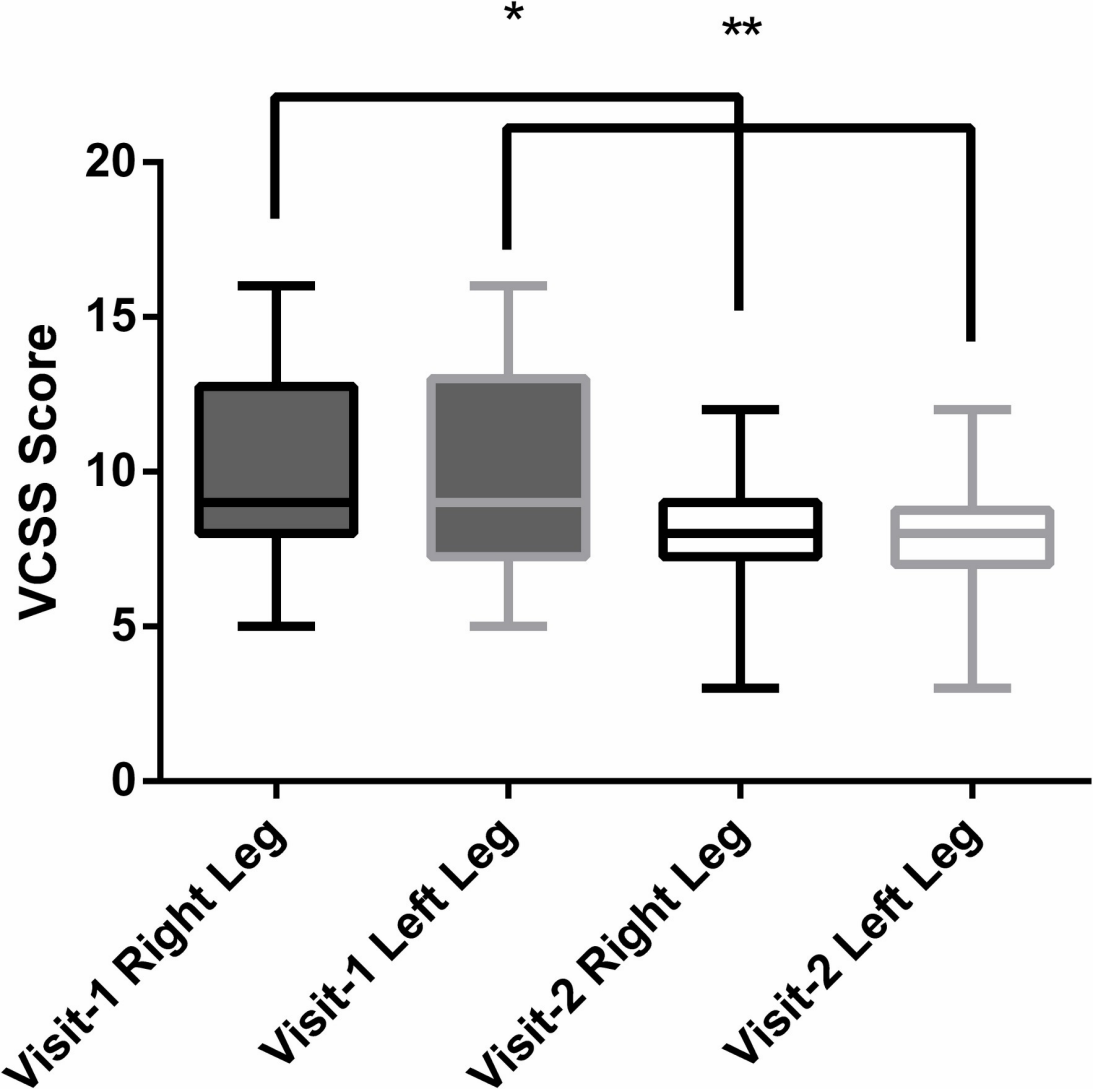
VCSS Scores before and after 6 weeks of treatment with the LivRelief varicose veins cream. **Mean Change in VCSS score of Left Leg between visits: *P =* <*0*.*0001*. *Mean Change in VCSS score of Right Leg between visits: *P = 0*.*0003*. Statistical analysis was completed using a paired t-test.

There was a significant improvement in the mean VCSS score per treated leg between visits 1 and 2, *P < 0*.*0001* and *P = 0*.*0003* for the left and right leg respectively (Table 3 & Fig 2).

#### Q-LES-Q-SF

The Q-LES-Q-SF was self-administered at visit 1 and again at visit 2.

**Fig 3:**
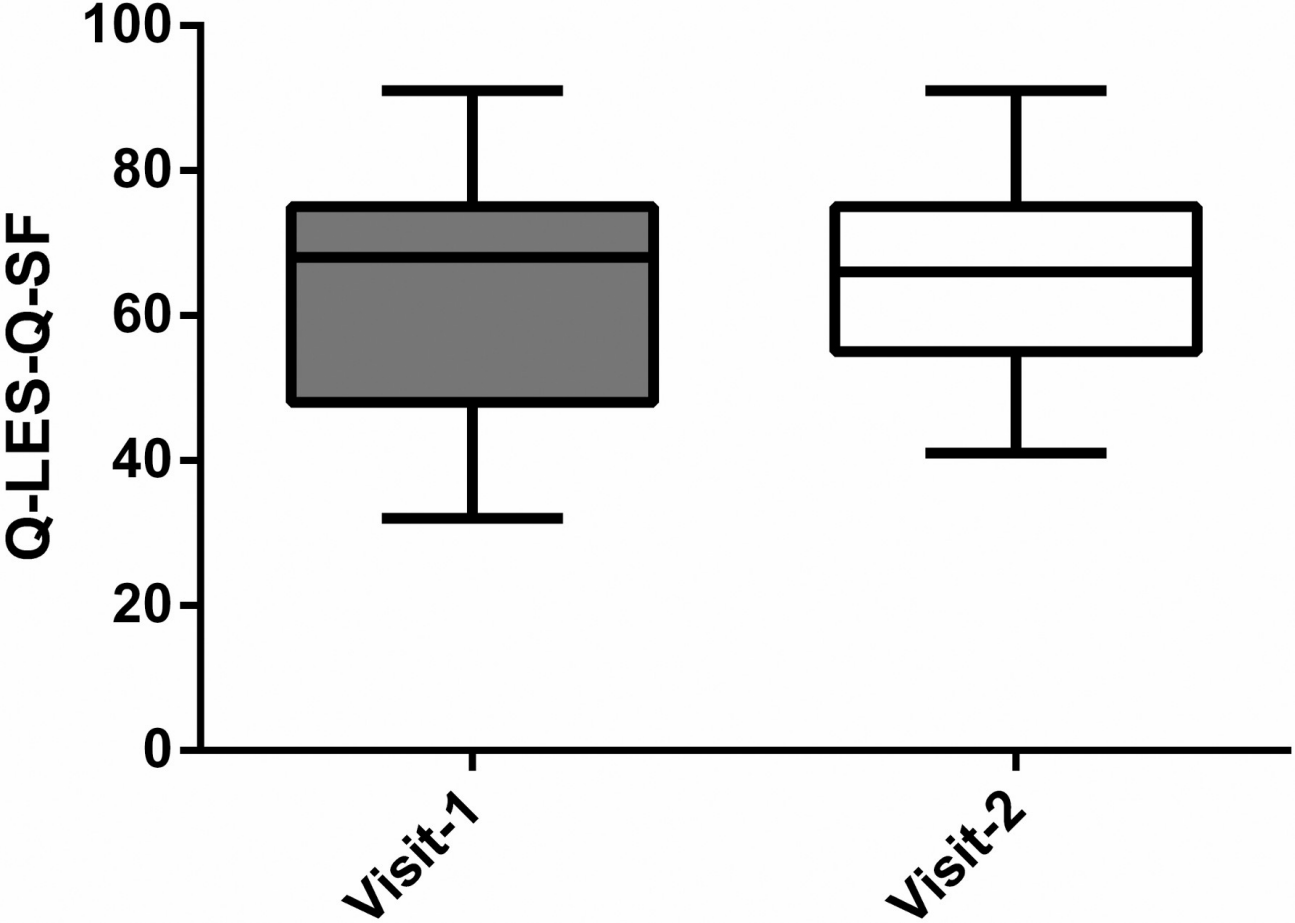
Q-LES-Q-SF Scores before and after 6 weeks of treatment with the LivRelief varicose veins cream.

There was no significant difference in the mean Q-LES-Q-SF score (Fig 3) between the study visits 1 & 2 (n = 30) at 63.1 and 65.8 respectively, *P = 0*.*246* (paired t-test).

#### CEAP classification for venous disease

**Fig 4:**
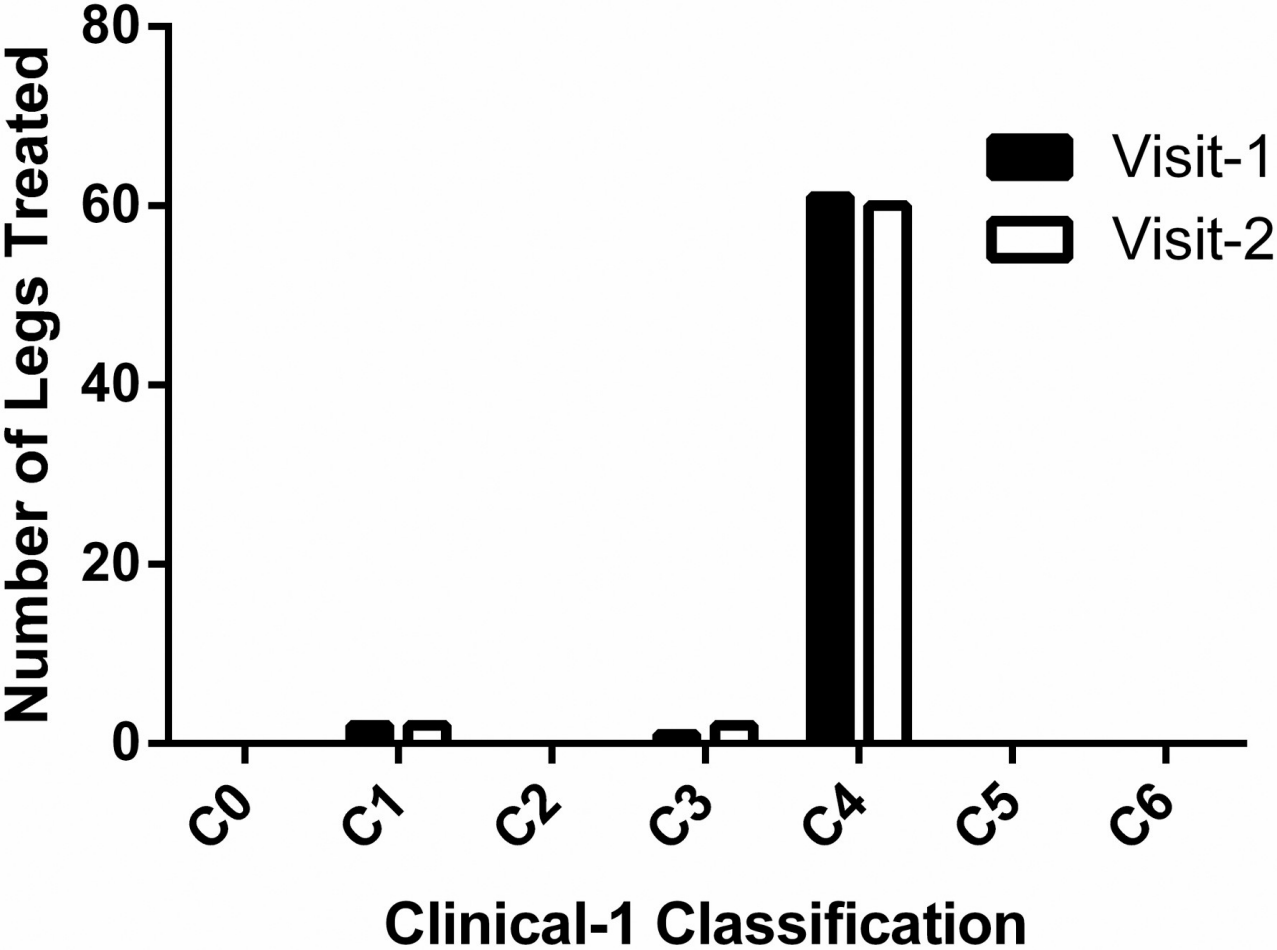
CEAP Classification before and after 6 weeks of treatment with the LivRelief varicose veins cream.

No change in CEAP classification was detected following treatment (Fig 4).

#### Circumferential measure of the index limb

The following locations were measured on each leg: The mid-foot, 5cm proximal to the medial malleoli and 5cm distal from the tibial tuberosity.

**Fig 5:**
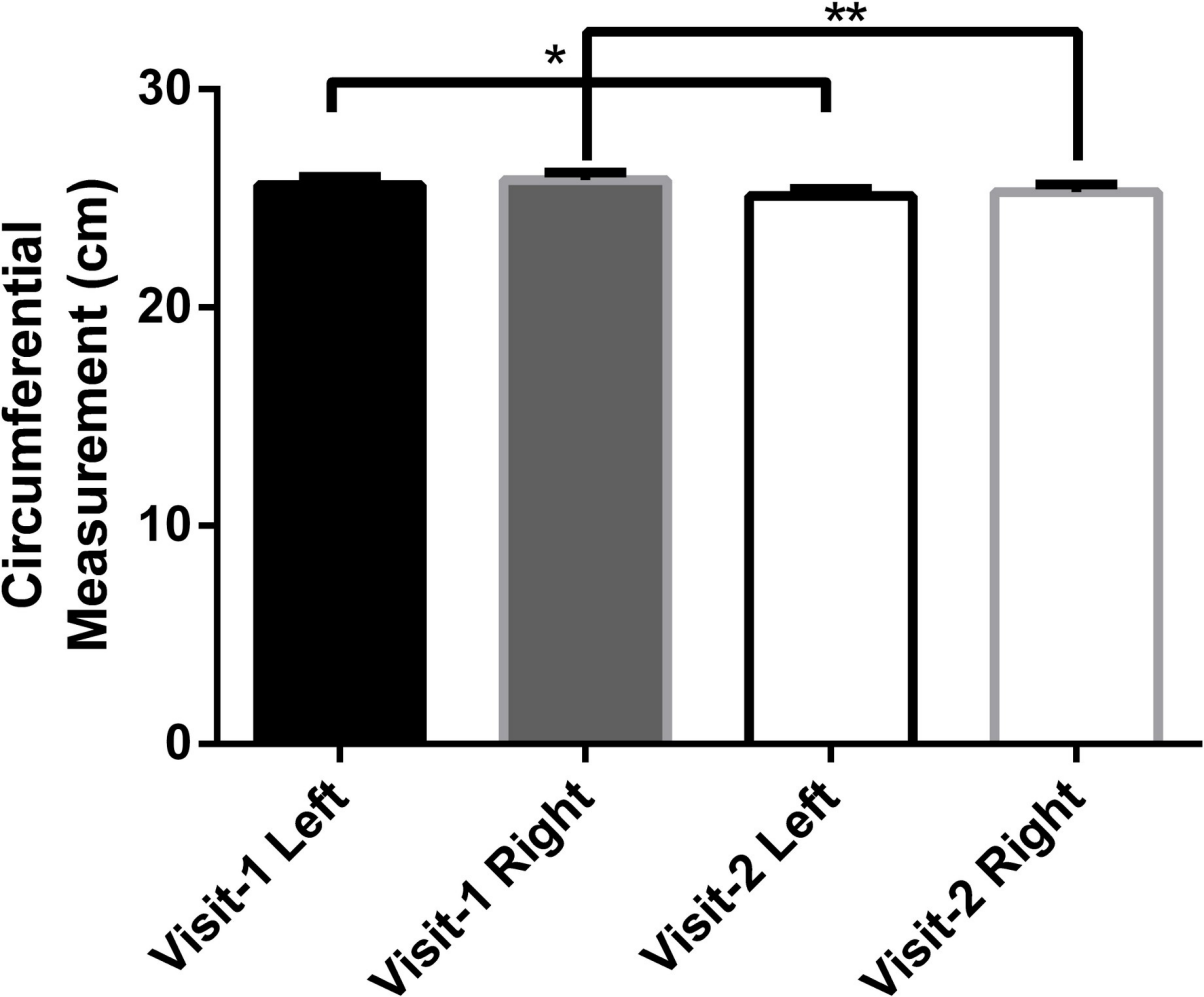
Mid-foot circumference before and after 6 weeks of treatment with the LivRelief varicose veins cream. Mean Change in Mid-Foot Circumference between visits 1 & 2 were analyzed using a paired t-test with *P =* 0.0017 and 0.0246 for the *Left and **Right legs respectively.

Analysis with a paired t-test showed a moderate reduction/improvement in the mean circumference of the midfoot between visits, *P = 0*.*0017* and *0*.*0246*, for the left and right foot respectively (Fig 5). However, there was no significant difference in the mean circumference of the locations: 5cm proximal to the Medial Malleoli or 5cm distal to the tibial tuberosity for either leg.

#### Adverse events

Two subjects developed blisters during treatment. One was determined to be “possibly related” and the other “unlikely to be related” to the use of the cream. The subject whose blisters were possibly related to the use of the cream was prematurely discontinued from treatment.

## Discussion

The VCSS was used to capture the physician’s evaluation of CVD symptoms in this study and the results, though inconclusive due to limitations in the study design, indicate that the cream may improve clinical symptoms when used as directed (Fig 2). In the current study, the Varicose Veins Cream resulted in a ~0.6-point reduction in the mean “*induration*” (a component of the VCSS) between baseline and 6 weeks of treatment, i.e.; [mean (SD)]: Right Leg: 0.8 (±1.0) to 0.3 (±0.8) and Left Leg: 0.9 (±1.1) to 0.3 (±0.8) with *P =* of 0.015 and 0.008, respectively. This observation, though not equivalent in significance, is comparable to that demonstrated by Pycnogenol, an oral VAD, tested in a similar population for a slightly longer duration [37] (Table 4). The mean (SD) total VCSS score per treated leg in the current study was 10.0 (±3) at baseline and 8.0 (±1.9) at 6 weeks, which is a score improvement of 2 points. This observation is comparable, though not equivalent in significance, to the efficacy of radiofrequency ablation (an invasive procedure) which had reported a score change of 3.42 at 3 months post treatment [38], see Table 4 below. The VCSS results of both referenced studies support the assertion that the implied improvement in clinical symptoms following the use of the varicose veins cream should be further investigated in a controlled experiment.

**Table 4:** VCSS as a measure of clinical efficacy in prior studies investigating the treatment of CVI.

Reference	Study summary	Baseline VCSS (Mean/ Median with SD/Range)	End of treatment VCSS (Mean/ Median with SD/Range)	Duration of Follow-up	Change over time
Belcaro, 2014 [16]	A CVI study that compared the efficacy of Pycnogenol (pine bark extract), Antistax (Red Vine Leaf extract) and compression Stockings over 8 weeks of treatment (n = 183). Pycnogenol was the only arm that showed a moderate but significant improvement in the mean VCSS score for induration in both legs from baseline.	Mean (SD)—Induration 2.3 (0.9)	Mean (SD)—Induration 1.4 (0.4)	8 weeks	0.9
Jin, et al., 2016 [38]	In a chart review of the treatment of varicose veins with radiofrequency ablation (n = 183) there was distinct clinical improvement in the mean pre-operative total VCSS score per leg at 3, 6 and 12 months post treatment.	Mean (SD) - 4.08 (±1.67)	Mean (SD)– 0.66 (±1.05), 0.58 (±1.02) and 0.63 (±1.14)	3, 6 & 12 months respectively	3.42, 3.5. 3.45 respectively

Though a comparison with some prior CVD studies does suggest that the Varicose Veins Cream may have a therapeutic effect, it has also highlighted a distinction between the current study population and many other prior clinical CVD investigations. Table 5 below lists 8 studies that investigated the therapeutic effect of a seven invasive CVD therapies and one pharmacological agent [39]. All 8 studies measured therapeutic change with the VCSS. The CEAP profile for seven of the eight study samples was comprised primarily of legs classified as ≤ C3, except for the study investigating Endovenous Laser Treatment (EVLT) & Radiofrequency Ablation (RFA). The sample for the Pycnogenol/Anistax/Compression study consisted of C3 to C4a limbs that were absent of pigmentation and inflammation at baseline [37]. By comparison, all the legs in the current observational study had pigmentation and 52% of them had signs of inflammation, both of which are characteristics of C4 (Fig 4). As such, the prevalence of CVI in the current sample is far greater than that of 7 of the 8 studies listed in Table 5. This difference makes it challenging to compare the current preliminary results with these studies to ascertain relative therapeutic potential. A future study should be conducted in a sub-sample of this population, C2-C3, with a positive control that has established efficacy within this subpopulation.

**Table 5:** Sample description from RCTs & chart reviews conducted in the CVD population.

Reference	Intervention	Baseline CEAP score	Baseline VCSS Score	Final VCSS Score	Score change	Follow-up
Vasquez & Munschauer, 2010[17]	saphenous vein RFA therapy.	C2:7%, C3:64%, C4:16%, C5:5% & C6:8%	8.8 (mean)	3.3 (mean)	3.6	4 Months
Vasquez & Munschauer, 2008[17]	stenting as treatment for occlusive lesions.	C2:23%, C3:65%, C4:8%, C5:2% and C6:2%.	8.5 (median)	2.0 (median)	6.5	12 months
Vasquez & Munschauer, 2008[17]	EVLT or radiofrequency ablation (RFA)	C3:37%, C4:30%, C5:12% & C6:20%.	11.5 (mean)	4.4 (mean)	7.1	3 months
*Belcaro et, al., 2014[16]	Pycnogenol (pine bark extract), Antistax (Red Vine Leaf extract) and compression Stockings	C3-C4a	Mean (SD)—Induration 2.3 (0.9)	Mean (SD)—Induration only 1.4 (0.4)	0.9	8 weeks
Jin et al., 2016[38]	radiofrequency ablation	C1:7%, C2:45%, C3:43% & C4:5%	Mean (SD) - 4.08 (±1.67)	Mean (SD): 0.66 (±1.05), 0.58 (±1.02) and 0.63 (±1.14)	3.42, 3.5. 3.45 respectively	3, 6 & 12 months respectively
Proebstle et al., 2018[40]	ablation of great saphenous vein (GSV) reflux by implantation of a PGA yarn was tested under ultrasound guidance	C2:54.3%, C3:27.2%, C4a:8.6%, C4b:4.9%, C5:2.5%, C6:2.5%	Mean (SD): 5.5 (±2.6)	Mean (SD): 1.6 (±1.9)	3.9	6 months
Gibson & Ferris, 2017[41]	Cyanoacrylate closure of incompetent great, small and accessory saphenous veins without the use of post-procedure compression	C2:36%, C3:28%, C4ab:34%, C5:2%,	Mean (SD): 6.5 (±2.4) (3–14)	Mean (SD): 1.8 (±1.4) (0–6)	4.7	30 days
Hartung, 2005[39]	iliocaval stenosis or occlusion	C2:23%, C3:65%, C4:8%, C5:2%, C6:2%	Median (SD): 8.5 (4–18)	Median (SD): 2 (0–9)	6.5	Median follow-up 27 months (2–103 months)

* Exclusion criteria included: Obesity, pigmentation, inflammation and the presence of ulcers, i.e.:”0” for VCSS questions: 4, 5 and 7–9. By comparison, in the Varicose Veins Study: all the legs had pigmentation (a characteristic of C4), 52% of the legs in the VVS study had signs of inflammation (a characteristic of C4).

In the current study, QoL was measured through the Q-LES-Q-SF and it showed no significant improvement from baseline (Fig 3). It has been recommended that both a generic and a disease specific QoL assessment be used when evaluating clinical improvement in CVD [1]. Due to their sensitivity to change, the addition of a venous disease specific QoL surveys, like the CIVIQ-20, would have been more likely to produce results consistent with those of the VCSS [34].

The Circumferential measurement of the lower limbs was performed to detect changes in peripheral edema before and after treatment with the varicose vein cream. Changes in edema have been evaluated in several placebo-controlled trials with statistically significant results [42, 43]. In this study, the mid-foot was the only location that showed a meaningful reduction in circumferential measure (Fig 5). However, the significance of these results in this design is tempered by the fact that in addition to CVD, there are a variety of medical conditions and associated medications that may cause lower limb edema [44]. Such comorbidities were not excluded from this study and there was no restriction on the use of concurrent medication. As a result, a change in the localized varicosity may not yield a concomitant change in edema. However, a significant change was detected in one of three measurement locations, the ‘mid-foot’ (Fig 5). This suggests that the measurement of edema may be a valuable objective assessment in future studies that employ appropriate eligibility criteria and restrictions that control for confounding variables. The results of the photographic assessment of skin discoloration were less impressive. 50% of the treated limbs showed a decrease in discoloration when compared to baseline with none showing an increase in discoloration. This assessment yields a subjective quantification of change and so its utility in a larger study of this product is questionable.

While there are multiple licensed natural health products that are indicated for the treatment of varicose veins, very few have undergone clinical testing to demonstrate product specific efficacy, as this is rarely a requirement for the issuance of a NPN. This pilot study produced SDs for the VCSS, circumferential measure of the treated leg and Q-LES-Q-SF that would be required to determine the sample size for a RCT in the same population. However, none of the prior studies reviewed in the post evaluation of the current study data used the VCSS as a *primary* outcome measure. While the VCSS does provide a dynamic option to assess therapeutic effect, the question of–*‘What is a meaningful treatment effect*?*’* remains. Other relevant questions are: **‘***What is a clinically meaningful reduction in ankle volume*?*’* And ‘*What is the best criteria for measuring the success of VAD treatment for varicose veins*?*’* [1]. These answers are required to determine an appropriate sample size that may include all CEAP clinical classifications.

## Conclusions

This observational pilot study was not designed to demonstrate statistical significance of a treatment effect, or even confirm clinical efficacy, but rather confirm that the selected sample could be successfully recruited, and the proposed data successfully collected from the target population. These feasibility objectives were achieved, and the resulting data suggests that the product could possibly improve some of the clinical symptoms associated with the presence varicose veins. The results from this study may eventually lead to a controlled investigation of the therapeutic effects of the Varicose Veins Cream. Confirmation and quantification of the efficacy of this product should be explored through a RCT with a design that minimizes the impact of confounding variables and measures key indicators of change in CVD severity.

## Supporting information

S1 Checklist TREND statement checklist. (PDF)

S1 Protocol Protocol Version 3 and 4 with ethics approvals. (PDF)
