# Supplementary material for: LivRelief varicose veins cream in the treatment of chronic venous insufficiency of the lower limbs: A 6-week single arm pilot study
Source: PLoS One. 2018 Dec 31;13(12):e0208954. doi: 10.1371/journal.pone.0208954 (PMC6312404; doi:10.1371/journal.pone.0208954)
Supplement: S1 Protocol — (PDF) [file pone.0208954.s002.pdf]

Title:
A Phase IV Interventional Pilot study of a licensed topical product for the treatment of varicose veins in adults.

Introduction/Context:
It is estimated that chronic venous disease in the lower extremities (e.g. varicose veins) is one of the most common pathological conditions in adults within industrialized countries, affecting ~40% of the population[1]. Chronic venous disease can encompass numerous pathologies whose casual factors can be both genetic and/or environmental and express a wide scope of symptomatic presentations from ulcers, deep vein thrombosis, and varicose veins[1]. The latter refers to a tortuous or twisted vein(s) that may be visibly enlarged or protrude physically due to the engorgement of blood within the peripheral lower limb vasculature. Mechanically, this deformation and poor blood flow is related to the reduction in tensile strength and function of veins leaflets that typically ensure a unidirectional movement of blood[2]. The loss of leaflet function causes blood reflux, localized pooling, and architectural obstructions which give rise to varicose veins.

Within Canada treatments for varicose veins can be segmented into two regulatory sections, the Therapeutic Products Directorate (TPD) that oversees drug and medical device usage and the Natural and Non-prescription Health Products Directorate (NNHPD) that oversees products deemed to originate from natural sources rather than human-derived innovations. With respect to NNHPD products and varicose veins, plant extracts from witch-hazel (*Hamamelis virginiana*)[3] and horse-chestnut (*Aesculus hippocastanum*)[4] are the primary natural medicinals used to treat this condition[5,6]. The sponsor (Delivra Inc.) currently holds a Natural Product Number (NPN) license for the manufacturing and sales of topical LivRelief™-Varicose (NPN 80029345) that includes 10% (w/w) witch-hazel[7]. In keeping with the vast majority of NPNs the efficacy of this hinges upon the ingredients-specific Health Canada monograph for topical witch-hazel[3]. This is the inverse to TPD drugs, which undergo rigorous product-specific testing to generate a product-specific monograph.

Recently, the NNHPD has signaled a broad reconstruction of its claim system to better distinguish those products that have undergone product-specific clinical testing as opposed to those which rely upon ingredient-specific monographs[8]. The underlying goal is to provide the Canadian consumer with sufficient scientifically-underpinned information and labeling to make informed purchases. With this in mind, the sponsor (Delivra Inc.) is interested in completing an study of its current product for its current claim within the scope and limitations as described by the product license[7]. In essence, a Phase-IV interventional pilot study is proposed.

Safety and Adverse Events

As licensed, if varicose veins persist or worsen after or during the course of treatment consult a healthcare practitioner, such as the Primary Investigator[7]. In rare cases the license product may cause a skin rash[7]. This rash is a known adverse event of the licensed product and use of the product should be discontinued immediately. More recently and not encompassed by the products licensing of federal monograph, several natural extracts (including witch-hazel) have been shown to cause contact dermatitis in some individuals[9]. As such, any acute or progressive skin inflammation is sufficient to warrant discontinuation of product use.

Absorption and Plasma Level:
To our knowledge there is no scientific information (peer-reviewed or otherwise) regarding the topical absorption, distribution, metabolism, or excretion of witch-hazel extract or a component thereof.

Study purpose and rational:
LivRelief Varicose Vein Cream is a Natural Health Product product licensed as a treatment for venous stasis (NPN 80029345). This phase IV interventional pilot study aims to collect information on specific objective and subjective measures by the sponsor. Quality of Life is measured through the Quality of Life Enjoyment and Satisfaction Questionnaire – Short Form (Q-LES-Q-SF). In combination and complementary to this questionnaire, the Venous Clinical Severity Score (VCSS) is employed whose construction was targeted directly at repeat longitudinal evaluations[12,13,14]. A preliminary dataset (N=30) is proposed in order to evaluate product use and data collection quality.

Description of the population to be studied (inclusion/exclusion criteria)
Once a signed consent is obtained, the participant is enrolled in the study and screened for the presence of varicose veins as defined by the chronic venous disorders Clinical-Etiology-Anatomy-Pathophysiology (CEAP) classification system[11,12] as (1) demonstrating a Varicose Veins score >0 at the prospective site of product application and (2) demonstrating an Active Ulcer Number equal to zero at the prospective site of product application.

Inclusion Criteria:
- Participants at least 19 years of age or older.
- Participants with lower limb varicose veins.

Exclusion Criteria
- Allergy to witch-hazel or any allergies in the cream.
- Intent to undergo surgical treatment varicose veins within the next six weeks.
- Pregnant or breastfeeding or planning to be pregnant.

- Any Dementia or Major Cognitive dysfunction that would preclude the individual's ability to provide informed consent or complete the Case Report Form.
- Any unstable medical condition (including but not limited to cardiovascular, cardiac/hypertension disease, moderate to severe kidney disease, and moderate to severe liver disease)
- Any medical condition that would preclude the participant's or a caregiver's ability to administer the product on a daily basis for the time period required to complete the study.
- An active ulcer at the site of product application (as evaluated during CEAP screening).

Participant Recruitment and Advertising:
Patients of the Mayer Institute suffering of varicose veins will be offered to participate in the study. Participants will also be recruited through advertisements (posters) at The Mayer Institute in Hamilton. The participant will contact the study investigator (they physician) and make an appointment to discuss the study and obtain consent. Each participant will be initially screened by a trained research coordinator, and then assessed by the principal investigator for health history and meeting of the inclusion criteria and ensure they do not meet any of the exclusion criteria (see below).

Study Design and Methodology:
The present study is designed as a phase IV interventional pilot study.

Statistical Analysis:
As described by Thabane et al. (2010)[15], we have assigned success criteria based on the following feasibility objectives:

- At least 70% of all eligible participants can be recruited
- Complete full data collection in at least 70% of all recruited participants

Dose and Mode of Administration:
Each day of the Experimental test period (6 weeks or 42 days) 1.0 mL (one pump~~s~~) of the product is applied to the varicose vein and surrounding area twice a day. This dosage is in alignment with the products licensed use[7].

Product and Ingredients' List:
The "LivRelief- Varicose Vein Cream" is being used for this study:

- NPN#: 80029345.
- List of ingredients:
  - **Active Ingredient**: Hamamelis virginiana L. (Witch Hazel) bark 10%

- **Non-Medicinal Ingredients**: Aesculus Hippocastanum (Horse Chestnut) Seed Extract, Arnica Montana Flower Extract, Black currant seed oil, Butylene Glycol, Carnauba Wax, Cetearyl olivate, Cetyl Palmitate, Chlorphenesin, Citrus limon extract, Dicaprylyl carbonate, Ethoxydiglycol, Glycerin, Goldenrod extract, Lecithin, Peucedanum ostruthium Leaf extract, Polyacrylate-13, Polyisobutene, Polysorbate 20, Purified water, Rosemary extract, Rubus idaeus (Raspberry) Seed oil, Ruscus Aculeatus Root Extract, Rutin, Sambucus nigra flower extract, Sorbitan Palmitate, Sorbitan olivate, Sunflower oil, Xanthan Gum, sodium phytate

Definition of endpoints:

Primary:

- Collection of data and completeness of datasets.

Exploratory analysis:

- Evaluation of statistical differences (if any) in objective and/or subjective measures at onset and completion of the trial.

Measurements and measurement instruments:

A combination of Objective and Subjective methods will be employed in this interventional study.

Objective measures:

- Circumferential measurements of the limb will be collected at onset and completion (twice) of the trial.
  Three landmarks will be used on the leg:
  - Mid foot
  - 5 cm proximal to the medial malleolus
  - 5 cm distal from the tibial tuberosity

- Photographic evidence of the discoloration of the skin in the vicinity of the varicose vein/limb along with incremental (metric) measure in frame. Photographic information will be collected at onset and completion (twice) of the trial.

Subjective measures:

- Clinical grade, Etiology, Anatomy, and Pathophysiology (CEAP) classification of the varicosity. The CEAP classification is assessed at onset and completion (twice) of the trial.
- Quality of Life Enjoyment and Satisfaction Questionnaire – Short Form (Q-LES-Q-SF) is recorded as Quality of Life questionnaire. The Q-LES-Q-SF is administered at onset and completion (twice) of the trial.

- Venous Clinical Severity Score (VCSS). The VCSS is assessed at onset and completion (twice) of the trial.
- Adverse events collection as self-reported incidents at the completion of trial and specific inquiries in regards to any adverse events at the site of administration, including rashes.

Schedule of Assessments

As described in Table 2, on Day 1 of the study the participant completes the Informed Consent Form (ICF), is evaluated according to inclusion and exclusion criteria, undergoes CEAP classification of the region in question, photographed within and limited to the region in question, limb diameter measurement, and Q-LES-Q-SF in this chronological order. On the same day and subsequent to these evaluations and measures the product is applied for the first time (Day 1) and materials and instructions are provided for self-administration over the proceeding period (41 days).

At the end of six weeks (42 days +/- 3 days) the same schedule of assessments is administered (with the exception of consent and inclusion/exclusion) as well as self-reported adverse events, if any.

Table 2 Graphical Schedule of Assessments

EVENT (maximum duration)	Screening and Baseline Visit 1 (Day 1)	Experimental Period (Day 2-40)	End Visit Visit 2 (Day 42)
Informed Consent Form (2 minutes)	✓		
Pregnancy Test (5 minutes)	✓		
Inclusion/Exclusion Criteria Evaluation (2 minutes)	✓		
Leg Circumferential measurement (5 minutes)	✓		✓
Skin Discoloration assessment (photography) (2 minutes)	✓		✓
CEAP Classification (5 minutes)	✓		✓
Quality of Life Enjoyment and Satisfaction Questionnaire – Short (Q-LES-Q-SF) (2 minutes)	✓		✓
Venous Clinical Severity Score (VCSS) (3 minutes)	✓		✓
Cream Application (twice a day) (<1 minute)	✓	✓	

Adverse Events Evaluation	✓	✓	✓

References:

1. Beebe-Dimmer, J. L., Pfeifer, J. R., Engle, J. S. & Schottenfeld, D. The epidemiology of chronic venous insufficiency and varicose veins. *Ann. Epidemiol.* **15,** 175–184 (2005).

2. Pascarella, L. & Schmid Schönbein, G. W. Causes of telengiectasias, reticular veins, and varicose veins. *Semin. Vasc. Surg.* **18,** 2–4 (2005).

3. Health Canada. Monograph - Topical Witch-hazel. at <http://webprod.hc-sc.gc.ca/nhpid-bdipsn/atReq.do?atid=witch.hazel.topical&lang=eng>

4. Health Canada. Monograph - Oral horsechestnut. at <http://webprod.hc-sc.gc.ca/nhpid-bdipsn/monoReq.do?id=118&lang=eng>

5. Foster, S. British Herbal Compendium: A Handbook of Scientific Information on Widely Used Plant Drugs. *HerbalGram* 74–76 (2008). at <https://login.ezproxy.endeavour.edu.au:2443/login?url=http://search.ebscohost.com/login.aspx?direct=true&db=awh&AN=35534078&site=eds-live&scope=site>

6. Mills, S. & Bone, K. *Principles & Practice of Phytotherapy--Modern Herbal Medicine*. *J. Altern. Complement. Med.* **7,** (2013).

7. Health Canada. Pharmax Varicose Natural Product Number License. at <https://health-products.canada.ca/lnhpd-bdpsnh/info.do?licence=80029345>

8. Canada, G. of. Consulting Canadians on the regulation of self-care products in Canada. at <https://www.canada.ca/en/health-canada/programs/consultation-regulation-self-care-products/consulting-canadians-regulation-self-care-products-canada.html>

9. Health Canada. Clinical Trials For Natural Health Products. at <http://www.hc-sc.gc.ca/dhp-mps/prodnatur/legislation/docs/clini_trials-essais_nhp-psn-eng.php>

10. Gangemi, S. *et al.* Contact dermatitis as an adverse reaction to some topically used European herbal medicinal products - Part 2: Echinacea purpurea-Lavandula angustifolia. *Contact Dermatitis* **72,** 193–205 (2015).

11. Klem, T. M. A. L., Sybrandy, J. E. M. & Wittens, C. H. A. Measurement of Health-related Quality of Life with the Dutch Translated Aberdeen Varicose Vein Questionnaire before and after Treatment. *Eur. J. Vasc. Endovasc. Surg.* **37,** 470–476 (2009).

12. Vasquez, M. A. *et al.* Revision of the venous clinical severity score: Venous outcomes consensus statement: Special communication of the American Venous Forum Ad Hoc Outcomes Working Group. *J. Vasc. Surg.* **52,** 1387–1396 (2010).

13. Kakkos, S. K. *et al.* Validation of the new venous severity scoring system in varicose vein surgery. *J. Vasc. Surg.* **38,** 224–228 (2003).

14. Marston, W. A. *et al.* Multicenter assessment of the repeatability and reproducibility of the revised Venous Clinical Severity Score (rVCSS). *J. Vasc. Surg. Venous Lymphat. Disord.* **1,** 219–224 (2013).

15. Thabane, L. *et al.* A tutorial on pilot studies: the what, why and how. *BMC Med Res Methodol* **10,** 1 (2010).

IRB INSTITUTIONAL REVIEW BOARD SERVICES

IRB APPROVAL WITH MODIFICATION / REB ATTESTATION

**DATE:**	20 Apr 2017
**TO:**	Perry Mayer, MD<br>The Mayer Institute
**PROTOCOL:**	Delivra Corp, A Phase IV Interventional Pilot study of a licensed topical product for the treatment of varicose veins in adults. (Pro00020990)
**SITE APPROVAL:**	20 Apr 2017
**EXPIRY DATE:**	20 Apr 2018

IRB APPROVED DOCUMENTATION

**Protocol Version:**	• V3 – April 2017
**Consent Form:**	• Informed Consent Form (IRB Services Approved Version 20 Apr 2017)
**Product Information:**	• Product Monograph: Health Canada Drugs and Health Products Hamamelis Water - Topical Hamamelis virginiana L. (Hamamelidaceae) (USDA 1994) Date Modified: 2017-01-10
**Recruitment Material:**	• Advertisement Poster "VARICOSE VEINS STUDY PARTICIPANTS NEEDED!" Version 1 – Apr. 2017
**Other Material:**	• Quality of Life Enjoyment and Satisfaction Questionnaire – Short Form (Q-LES-Q-SF) Not Dated<br>• VENOUS CLINICAL SEVERITY SCORE (VCSS) Not Dated

The IRB reviewed and granted approval with the modifications listed below:

- **Modification to the Informed Consent Form**
- **Modification to the Advertisement Poster**

If you wish to have the IRB reconsider the imposed modifications, you may follow the procedures outlined below:

1. Submit supporting documentation that addresses the IRB’s concerns.
2. Provide a written justification for relief of any IRB imposed condition.

irbservices.com

372 Hollandview Trail | Suite 300 | Aurora, Ontario, Canada L4G 0A5
Office: 905.727.7989 | Fax: 905.727.7990

You have been unconditionally approved as the Qualified Investigator at your clinical trial site, as defined in Health Canada regulations, for the above study.

Membership List / Investigator Responsibilities

You can access the most recent IRB membership list and a copy of the General Guidance: Investigator Responsibilities by going to your CIRBI homepage (“My Home”) and selecting “Chesapeake IRB Home,” then selecting the “IRB Services Reference Materials.”

Your responsibilities to the IRB include, but are not limited to, informing the IRB of the following using CIRBI:

- Modifications to research (e.g., protocol amendments, revised consent forms, new or revised subject recruitment materials, change of site information, change of investigator)
- Prompt Reporting Events (e.g., protocol deviations, serious unexpected adverse reactions, unanticipated problems)
- Continuing review/termination reports (i.e., progress reports)

Compliance Statement/Attestation

IRB Services attests that the protocol and consent document have been approved, as described above, and the membership of the IRB complies with the requirements defined in Health Canada regulations, 21 CFR parts 56 and 312.3 and 45 CFR 46. The IRB carries out its functions in accordance with good clinical practices (e.g., ICH GCP Guidelines) and Health Canada regulations and in compliance with FDA 21 CFR parts 50 and 56, DHHS 45 CFR part 46, and the Tri-Council Policy Statement for Ethical Conduct of Research Involving Humans, as appropriate to the research.

The IRBs of IRB Services are registered with OHRP and FDA as follows:
- ON IRB registration #IRB00000776
- QC IRB registration #IRB00005290

If you have any questions, please do not hesitate to contact us at 905-727-7989 or via Contact IRB in CIRBI.

Thank you for selecting IRB Services to review your research project.

Sincerely,
Institutional Review Board Services

Michael Burdo
Coordinator
Client Services

irbservices.com
372 Hollandview Trail | Suite 300 | Aurora, Ontario, Canada L4G 0A5
Office: 905.727.7989 | Fax: 905.727.7990

Title:
A Phase IV Interventional Pilot study of a licensed topical product for the treatment of varicose veins in adults.

Introduction/Context:
It is estimated that chronic venous disease in the lower extremities (e.g. varicose veins) is one of the most common pathological conditions in adults within industrialized countries, affecting ~40% of the population[1]. Chronic venous disease can encompass numerous pathologies whose casual factors can be both genetic and/or environmental and express a wide scope of symptomatic presentations from ulcers, deep vein thrombosis, and varicose veins[1]. The latter refers to a tortuous or twisted vein(s) that may be visibly enlarged or protrude physically due to the engorgement of blood within the peripheral lower limb vasculature. Mechanically, this deformation and poor blood flow is related to the reduction in tensile strength and function of veins leaflets that typically ensure a unidirectional movement of blood[2]. The loss of leaflet function causes blood reflux, localized pooling, and architectural obstructions which give rise to varicose veins.

Within Canada treatments for varicose veins can be segmented into two regulatory sections, the Therapeutic Products Directorate (TPD) that oversees drug and medical device usage and the Natural and Non-prescription Health Products Directorate (NNHPD) that oversees products deemed to originate from natural sources rather than human-derived innovations. With respect to NNHPD products and varicose veins, plant extracts from witch-hazel (*Hamamelis virginiana*)[3] and horse-chestnut (*Aesculus hippocastanum*)[4] are the primary natural medicinals used to treat this condition[5,6]. The sponsor (Delivra Inc.) currently holds a Natural Product Number (NPN) license for the manufacturing and sales of topical LivRelief™-Varicose (NPN 80029345) that includes 10% (w/w) witch-hazel[7]. In keeping with the vast majority of NPNs the efficacy of this hinges upon the ingredients-specific Health Canada monograph for topical witch-hazel[3]. This is the inverse to TPD drugs, which undergo rigorous product-specific testing to generate a product-specific monograph.

Recently, the NNHPD has signaled a broad reconstruction of its claim system to better distinguish those products that have undergone product-specific clinical testing as opposed to those which rely upon ingredient-specific monographs[8]. The underlying goal is to provide the Canadian consumer with sufficient scientifically-underpinned information and labeling to make informed purchases. With this in mind, the sponsor (Delivra Inc.) is interested in completing an study of its current product for its current claim within the scope and limitations as described by the product license[7]. In essence, a Phase-IV interventional pilot study is proposed.

Safety and Adverse Events

As licensed, if varicose veins persist or worsen after or during the course of treatment consult a healthcare practitioner, such as the Primary Investigator[7]. In rare cases the license product may cause a skin rash[7]. This rash is a known adverse event of the licensed product and use of the product should be discontinued immediately. More recently and not encompassed by the products licensing of federal monograph, several natural extracts (including witch-hazel) have been shown to cause contact dermatitis in some individuals[9]. As such, any acute or progressive skin inflammation is sufficient to warrant discontinuation of product use.

Absorption and Plasma Level:
To our knowledge there is no scientific information (peer-reviewed or otherwise) regarding the topical absorption, distribution, metabolism, or excretion of witch-hazel extract or a component thereof.

Study purpose and rational:
LivRelief Varicose Vein Cream is a Natural Health Product licensed as a treatment for venous stasis (NPN 80029345). This phase IV interventional pilot study aims to collect information on specific objective and subjective measures by the sponsor. Quality of Life is measured through the Quality of Life Enjoyment and Satisfaction Questionnaire – Short Form (Q-LES-Q-SF). In combination and complementary to this questionnaire, the Venous Clinical Severity Score (VCSS) is employed whose construction was targeted directly at repeat longitudinal evaluations[12,13,14]. A preliminary dataset (N=30) is proposed in order to evaluate product use and data collection quality.

Description of the population to be studied (inclusion/exclusion criteria)
Once a signed consent is obtained, the participant is enrolled in the study and screened for the presence of varicose veins as defined by the chronic venous disorders Clinical-Etiology-Anatomy-Pathophysiology (CEAP) classification system[11,12] as (1) demonstrating a Varicose Veins score >0 at the prospective site of product application and (2) demonstrating an Active Ulcer Number equal to zero at the prospective site of product application.

Inclusion Criteria:
- Participants at least 19 years of age or older.
- Participants with lower limb varicose veins.

Exclusion Criteria
- Allergy to witch-hazel or any allergies in the cream.
- Intent to undergo surgical treatment varicose veins within the next six weeks.
- Pregnant or breastfeeding or planning to be pregnant.

- Any Dementia or Major Cognitive dysfunction that would preclude the individual's ability to provide informed consent or complete the Case Report Form.
- Any unstable medical condition (including but not limited to cardiovascular, cardiac/hypertension disease, moderate to severe kidney disease, and moderate to severe liver disease)
- Any medical condition that would preclude the participant's or a caregiver's ability to administer the product on a daily basis for the time period required to complete the study.
- An active ulcer at the site of product application (as evaluated during CEAP screening).

Participant Recruitment and Advertising:
Patients of the Mayer Institute suffering of varicose veins will be offered to participate in the study. Participants will also be recruited through advertisements (posters) at The Mayer Institute in Hamilton. The participant will contact the study investigator (they physician) and make an appointment to discuss the study and obtain consent. Each participant will be initially screened by a trained research coordinator, and then assessed by the principal investigator for health history and meeting of the inclusion criteria and ensure they do not meet any of the exclusion criteria (see below).

Study Design and Methodology:
The present study is designed as a phase IV interventional pilot study.

Statistical Analysis:
As described by Thabane et al. (2010)[15], we have assigned success criteria based on the following feasibility objectives:

- At least 70% of all eligible participants can be recruited
- Complete full data collection in at least 70% of all recruited participants

Dose and Mode of Administration:
Each day of the Experimental test period (6 weeks or 42 days) 0.8 mL (one pump) of the product is applied to the varicose vein and surrounding area twice a day. This dosage is in alignment with the products licensed use[7].

Product and Ingredients' List:
The "LivRelief- Varicose Vein Cream**"** is being used for this study:

- NPN#: 80029345.
- List of ingredients:
  - **Active Ingredient**: Hamamelis virginiana L. (Witch Hazel) bark 10%

- **Non-Medicinal Ingredients**: Aesculus Hippocastanum (Horse Chestnut) Seed Extract, Arnica Montana Flower Extract, Black currant seed oil, Butylene Glycol, Carnauba Wax, Cetearyl olivate, Cetyl Palmitate, Chlorphenesin, Citrus limon extract, Dicaprylyl carbonate, Ethoxydiglycol, Glycerin, Goldenrod extract, Lecithin, Peucedanum ostruthium Leaf extract, Polyacrylate-13, Polyisobutene, Polysorbate 20, Purified water, Rosemary extract, Rubus idaeus (Raspberry) Seed oil, Ruscus Aculeatus Root Extract, Rutin, Sambucus nigra flower extract, Sorbitan Palmitate, Sorbitan olivate, Sunflower oil, Xanthan Gum, sodium phytate

Definition of endpoints:

Primary:

- Collection of data and completeness of datasets.

Exploratory analysis:

- Evaluation of statistical differences (if any) in objective and/or subjective measures at onset and completion of the trial.

Measurements and measurement instruments:

A combination of Objective and Subjective methods will be employed in this interventional study.

Objective measures:

- Circumferential measurements of the limb will be collected at onset and completion (twice) of the trial.
  Three landmarks will be used on the leg:
  - Mid foot
  - 5 cm proximal to the medial malleolus
  - 5 cm distal from the tibial tuberosity

- Photographic evidence of the discoloration of the skin in the vicinity of the varicose vein/limb along with incremental (metric) measure in frame. Photographic information will be collected at onset and completion (twice) of the trial.

Subjective measures:

- Clinical grade, Etiology, Anatomy, and Pathophysiology (CEAP) classification of the varicosity. The CEAP classification is assessed at onset and completion (twice) of the trial.
- Quality of Life Enjoyment and Satisfaction Questionnaire – Short Form (Q-LES-Q-SF) is recorded as Quality of Life questionnaire. The Q-LES-Q-SF is administered at onset and completion (twice) of the trial.

- Venous Clinical Severity Score (VCSS). The VCSS is assessed at onset and completion (twice) of the trial.
- Adverse events collection as self-reported incidents at the completion of trial and specific inquiries in regards to any adverse events at the site of administration, including rashes.

Schedule of Assessments
As described in Table 2, on Day 1 of the study the participant completes the Informed Consent Form (ICF), is evaluated according to inclusion and exclusion criteria, undergoes CEAP classification of the region in question, photographed within and limited to the region in question, limb diameter measurement, and Q-LES-Q-SF in this chronological order. On the same day and subsequent to these evaluations and measures the product is applied for the first time (Day 1) and materials and instructions are provided for self-administration over the proceeding period (41 days).

At the end of six weeks (42 days +/- 3 days) the same schedule of assessments is administered (with the exception of consent, pregnancy test and inclusion/exclusion) as well as self-reported adverse events, if any.

Table 2 Graphical Schedule of Assessments

**EVENT (maximum duration)**	**Screening and Baseline Visit 1 (Day 1)**	**Experimental Period (Day 2-40)**	**End Visit Visit 2 (Day 42)**
Informed Consent Form (2 minutes)	✓		
***Pregnancy Test (5 minutes)	✓		
Inclusion/Exclusion Criteria Evaluation (2 minutes)	✓		
Leg Circumferential measurement (5 minutes)	✓		✓
Skin Discoloration assessment (photography) (2 minutes)	✓		✓
CEAP Classification (5 minutes)	✓		✓
Quality of Life Enjoyment and Satisfaction Questionnaire – Short (Q-LES-Q-SF) (2 minutes)	✓		✓
Venous Clinical Severity Score (VCSS) (3 minutes)	✓		✓
Cream Application (twice a day) (<1 minute)	✓	✓	

Adverse Events Evaluation	✓	✓	✓

***A pregnancy test is not required for female subjects of “non-childbearing potential”, i.e.: sterile or post-menopausal. Female subjects will state their reproductive status at screening.**

References:

1. Beebe-Dimmer, J. L., Pfeifer, J. R., Engle, J. S. & Schottenfeld, D. The epidemiology of chronic venous insufficiency and varicose veins. *Annals of Epidemiology* **15,** 175–184 (2005).
2. Pascarella, L. & Schmid Schönbein, G. W. Causes of telengiectasias, reticular veins, and varicose veins. *Semin. Vasc. Surg.* **18,** 2–4 (2005).
3. Health Canada. Monograph - Topical Witch-hazel.
4. Health Canada. Monograph - Oral horsechestnut.
5. Foster, S. British Herbal Compendium: A Handbook of Scientific Information on Widely Used Plant Drugs. *HerbalGram* 74–76 (2008).
6. Mills, S. & Bone, K. *Principles & Practice of Phytotherapy--Modern Herbal Medicine*. *Journal of Alternative & Complementary Medicine* **7,** (2013).
7. Health Canada. Pharmax Varicose Natural Product Number License.
8. Canada, G. of. Consulting Canadians on the regulation of self-care products in Canada.
9. Gangemi, S. *et al.* Contact dermatitis as an adverse reaction to some topically used European herbal medicinal products - Part 2: Echinacea purpurea-Lavandula angustifolia. *Contact Dermatitis* **72,** 193–205 (2015).

IRB APPROVAL/REB ATTESTATION

MOD00211512

**DATE:**	30 May 2017
**TO:**	Perry Mayer, MD<br>The Mayer Institute
**PROTOCOL:**	Delivra Corp - A Phase IV Interventional Pilot study of a licensed topical product for the treatment of varicose veins in adults (Pro00020990)
**APPROVAL DATE:**	30 May 2017

IRB APPROVED DOCUMENTATION

Documentation:

- Amended Protocol V4 dated May 2017 (Clean & Track Changes)
- Summary of Changes to study protocol Date: 29 May 2017

The IRB reviewed and approved the above referenced documentation.

Current IRB Approved Consent Document

The IRB determined there were no changes required to the current Consent Form. Please continue using the Consent Form electronically available on your CIRBI workspace under the "IRB Issued Documents" tab.

Membership List

You can access a copy of the most recent IRB membership list by going to your CIRBI homepage ("My Home") and selecting "Chesapeake IRB Home," then selecting "IRB Services Reference Materials."

Investigator Responsibilities

Your responsibilities are defined in pertinent regulations, ICH GCP Guidelines as well as the General Guidance: Investigator Responsibilities. You can access a copy of the General Guidance: Investigator Responsibilities by going to your CIRBI homepage ("My Home") and selecting "Chesapeake IRB Home," then selecting "IRB Services Reference Materials."

Your responsibilities to the IRB include, but are not limited to, informing the IRB of the following using CIRBI:

- Modifications to research (e.g., protocol amendments, revised consent forms, new or revised subject recruitment materials, change of site information, change of investigator)
- Prompt Reporting Events (e.g., protocol deviations, serious unexpected adverse reactions, unanticipated problems)
- Continuing review/termination reports (i.e., progress reports)

372 Hollandview Trail | Suite 300 | Aurora, Ontario, Canada L4G 0A5
irbservices.com | Office: 905.727.7989 | Fax: 905.727.7990

<u>Compliance Statement/Attestation</u>

IRB Services attests that the above document(s) have been approved, as described above, and the membership of the IRB complies with the requirements defined in Health Canada regulations, 21 CFR parts 56 and 312.3 and 45 CFR 46. The IRB carries out its functions in accordance with good clinical practices (e.g., ICH GCP Guidelines) and Health Canada regulations and in compliance with FDA 21 CFR parts 50 and 56, DHHS 45 CFR part 46, and the Tri-Council Policy Statement for Ethical Conduct of Research Involving Humans, as appropriate to the research.

The IRBs of IRB Services are registered with OHRP and FDA as follows:

- ON IRB registration #IRB00000776
- QC IRB registration #IRB00005290

If you have any questions, please do not hesitate to contact us at 905-727-7989 or via Contact IRB in CIRBI.

Thank you for selecting IRB Services to review your research project.

Sincerely,
Institutional Review Board Services

Michael Burdo
Coordinator
Client Services

Association for the Accreditation of Human Research Protection Programs, Inc. Full Accreditation

372 Hollandview Trail | Suite 300 | Aurora, Ontario, Canada L4G 0A5
irbservices.com | Office: 905.727.7989 | Fax: 905.727.7990
